# Enhancing human spatial awareness through augmented reality technologies

**DOI:** 10.1007/s13534-025-00502-7

**Published:** 2025-09-08

**Authors:** Janka Hatvani, Dominik Csatári, Márton Áron Fehér, Ágoston Várhidy, Jongmo Seo, György Cserey

**Affiliations:** 1 Faculty of Information Technology and Bionics, Pázmány Péter Catholic Unviersity, 50/a Práter utca, Budapest, 1083 Hungary; 2 Department of Electrical and Computer Engineering, Seoul National University, 1 Gwanak-ro, Seoul, 08826 South Korea

**Keywords:** Spatial awareness, Deep learning, Augmented reality, Sonar

## Abstract

Augmented reality (AR) has emerged as a powerful tool for enhancing human spatial awareness by overlaying digital information onto the physical world. This paper presents a review of the methodologies that enable AR-based spatial perception, with a focus on challenging environments such as underwater and disaster scenarios. We review state-of-the-art deep learning approaches for 3D data interpretation and completion, including voxel-based, point-based, and view-based methods. As part of this review, we implement an AR-enabled spatial awareness system, where the investigated deep learning solutions can be tested directly. In our approach, a robotic arm with an ultrasound sensor performs 2D scans underwater, from which a 3D point cloud of the scene is reconstructed. Using the reviewed deep learning networks, the point cloud is segmented in order to identify objects of interest, and point cloud completion is performed to infer missing structure. We report experimental results from synthetic data and underwater scanning trials, demonstrating that the system can recover and augment unseen spatial information for the user. We discuss the outcomes, including segmentation accuracy and completeness of reconstructions, as well as challenges such as data scarcity, noise, and real-time constraints. The paper concludes that, when combined with robust sensing and 3D deep learning techniques, AR enhances human spatial awareness in environments where direct perception is limited. The need for more adequate metrics to describe point clouds and for more labeled sonar datasets is discussed.

## Introduction

**Fig. 1:**
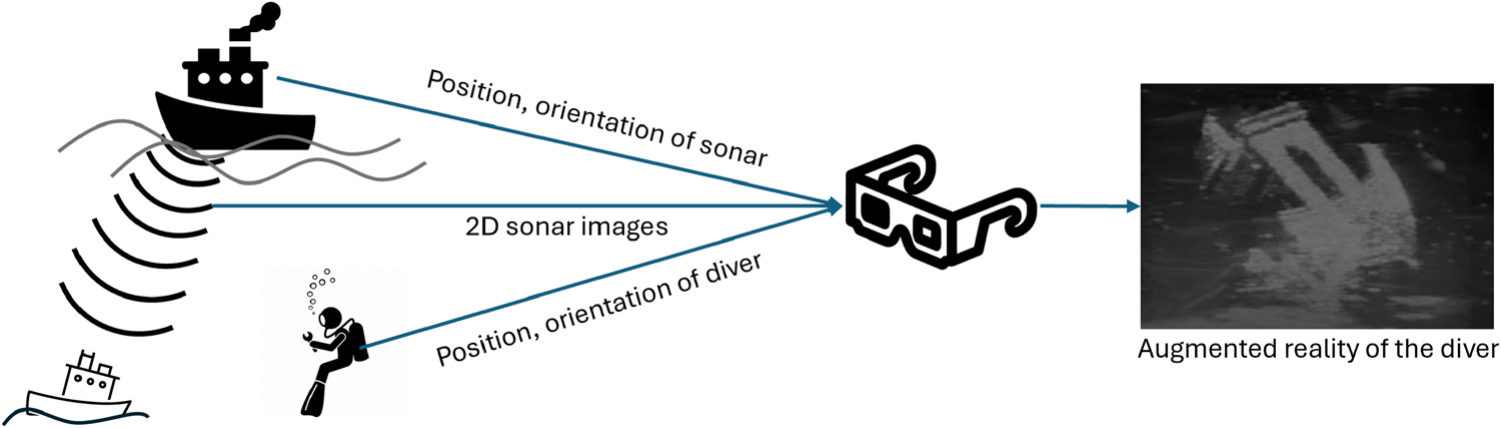
AR system utilized to augment the spatial awareness of a diver during a rescue mission. Sonar images of a sunken ship are transmitted with position and orientation information of the sonar and the diver to the AR system. A three-dimensional representation of a sonar image is constructed and rendered from the perspective of the diver

Human spatial awareness—the understanding of our surroundings and the spatial relationships within them—is critical in many tasks and environments. Augmented Reality (AR) technologies, which overlay computer-generated information onto the user perception of the real world, have been shown to improve spatial reasoning and learning outcomes [1, 2] (Fig. 1).

By superimposing crucial sensor data or navigational cues onto the view of the user, AR can help overcome human perceptual limits. For instance, firefighters operating in smoke-filled buildings can benefit from AR helmets that display building layouts or the locations of victims using thermal imaging, thereby greatly improving their situational awareness [3]. Similarly, in underwater domains where visibility is extremely limited, AR can assist divers by integrating sonar readings into a visual heads-up display. A notable example is the diver augmented vision display from the U.S. Navy, a helmet-mounted AR system that projects pre-acquired sonar scans or live acoustic camera feeds as a composite 3D map to the diver, helping view in dark or turbid waters [4]. Realizing these capabilities requires a confluence of appropriate imaging sensors and robust 3D reconstruction, mapping techniques. Interpretation algorithms, often powered by machine learning, are crucial to filter, classify, or complete sensor-derived data. Finally, the AR display and interface must be designed to effectively integrate this information into the user perception without causing mental overload.

Advances in sonar technology (e.g., higher resolution transmit-receivers) and processing have improved underwater 3D mapping. Researchers have developed SLAM (simultaneous localization and mapping) algorithms for sonar-equipped AUVs: each 2D sonar image can be back-projected into 3D space if the sensor position and orientation are tracked [5]. Merging many such 3D projections can yield a sparse but cumulative point cloud of the scene, allowing to map complex 3D environments like shipwrecks or cave interiors. However, sonar reconstructions still face limitations: the data tend to be sparse and noisy, and small or soft targets (e.g., a human body or a thin cable) produce weak echoes that are easily lost [6].

Deep learning has significantly advanced the state-of-the-art in processing 3D point clouds. Three major families of deep learning approaches are voxel-based convolutional neural networks (CNNs), point-based networks, and view-based or multi-view methods. Each offers different advantages for interpreting (classification tasks) or enhancing (completing missing parts, or increasing the number of points for a more appealing visualization) spatial data for AR.

Early attempts to apply CNNs to 3D data often transformed point clouds or meshes into a voxel grid, allowing the use of standard 3D convolutions to learn features for classification tasks [7] or object reconstruction and completion [8]. However, a key limitation of such methods is resolution: a grid must be relatively coarse (e.g., $32^3$ or $64^3$ voxels) to be computationally feasible. This causes the loss of fine details and high memory usage in tasks where images are sparse with a high ratio of background elements. Subsequent improvements, like octree-based CNNs and sparse convolutions, have alleviated this to some extent by focusing computation on occupied regions, but the trade-off between detail and efficiency remains [9, 10].

An alternative was provided with *PointNet* [11], which introduced a novel architecture to process these unordered point sets directly. PointNet uses multilayer perceptrons (MLPs) to independently transform the coordinates of each point into a higher-dimensional space and then aggregate these point-wise features into a global feature. This global feature can be used for classification or further processed in segmentation tasks. PointNet++ [12] also captures local geometric relations, by applying transformations in a local neighborhood around each point, on multiple scales. This hierarchical approach significantly improved performance, especially in scenes with non-uniform point densities or complex geometries. It has also been reported to accurately classify even sparse sonar point clouds [13]. Point-based solutions have also been used for point completion. The point completion network [14] uses a PointNet-based encoder, while the variational relational point completion network [15] introduced probabilistic mapping and relational feature learning. Graph convolution is another example of establishing local connections in [16]. A limiting factor of the point-based methods is the fixed number of network inputs. This means that larger point clouds need to be processed in batches and stitched together [17].

A third paradigm for processing 3D point clouds takes multiple 2D views or projections of the data and feeds these to 2D CNNs. The enhanced images are back-projected into 3D and merged to produce a completed point cloud [18, 19]. This approach allows for dynamic point allocation based on image content, enabling it to produce a variable number of points and finer detail where necessary. These methods also tend to handle large missing regions better by enforcing consistency from multiple angles. These aspects align well with how humans recognize objects. PIXOR [20] uses a compact 2D representation of a single bird’s eye view for object detection. LU-Net [21] combines view- and point-based methods for classification tasks, first detecting 3D features in the point cloud and then projecting them to 2D for further processing.

In summary, AR systems stand to gain from these advances: deep models can segment and label parts of the environment, ensuring that pats of the scene are correctly identified. They can also predict what might lie in sensor blind spots, allowing AR to display estimations of occluded structures. A challenge in training these models is the lack of large labeled datasets for acoustic images, hindering the development of learned enhancements or detection algorithms [6].

**Fig. 2:**
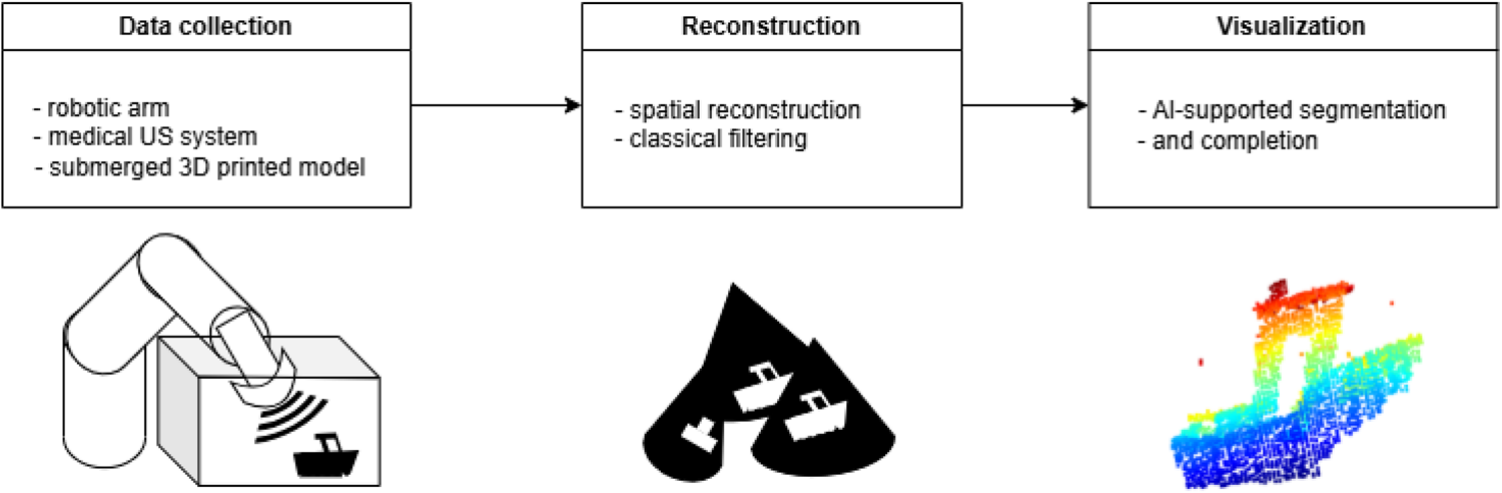
Overall pipeline of the AR-enabled underwater scanning system

In the following sections, this paper presents a framework for an underwater exploration AR pipeline (Fig. 2), in which the reviewed deep learning techniques are tested to produce visually enhanced acoustic point clouds of the underwater scene. We then present experimental results from this system, evaluate its performance (segmentation accuracy, reconstruction completeness), and discuss the challenges encountered (such as sensor noise, limited training data, and real-time processing demands). Finally, we conclude by reflecting on how these developments can be integrated into AR systems to tangibly enhance human spatial awareness, and we outline directions for future research.

## Methods

The following section describes the framework where the reviewed algorithms were tested for AR-enabled spatial awareness. In this setup, a robotic arm equipped with an ultrasound imaging sensor performs scans to create a 3D map of an underwater scene. We describe the methodology for generating a point cloud from multiple 2D ultrasound images, segmenting that point cloud using a retrained PointNet model to isolate objects of interest, and then applying a multi-view point cloud completion technique to produce a more complete model of those objects.

### Ultrasound scanning with a robotic arm

**Fig. 3:**
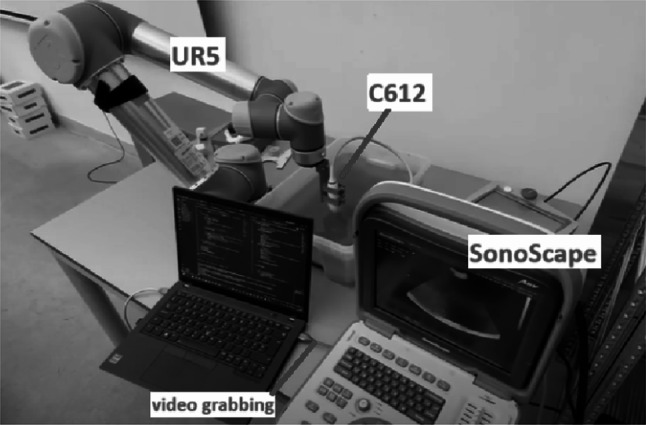
The experimental setup. An A6V Sonoscape US machine with a microconvex C612 B-mode transducer is mounted on the UR5 robotic arm, using a custom-printed plastic bracket. The robotic arm communicated with the laptop through an ethernet cable, while the analogue VGA output of the Sonoscape screen was grabbed using an A/D converter

The setup shown in Fig. 3 uses an UR5 robotic arm (Universal Robots, Boston, MA, USA) mounted on a stable platform to carry the ultrasound imaging sensor, a microconvex C612 transducer, connected to a veterinary A6V Sonoscape ultrasound machine (SonoScape, Shenzhen, China) that produces a 2D fan-shaped acoustic image (central frequency of 6.5 MHz, field of view 96°). As multibeam sonars typically operate at frequencies between 70 and 700 kHz, the scene was scaled to match the short wavelength of the ultrasound machine. A plastic tank measuring 0.4 m x 0.6 m x 0.15 m was filled with tap water. Small gravel was used to simulate the riverbed and talc powder was used to achieve turbidity. As target objects, 3D-printed PLA models (ship, airplane) were laid on the bottom of the tank.

A software system was developed in Python, focusing on synchronizing the motion of the robotic arm with the ultrasonic data acquisition. After calibration the system logs positional data from the robotic arm, ensuring that the position and orientation of the image plane in a global coordinate system is known. The robotic arm executes a scanning pattern to sweep the ultrasound beam around the area of interest. In our experiments a grid sweep was performed, moving the sensor in a plane parallel to the bottom of the tank, and then at a couple of different angles to cover different views. At each scan position, a 2D ultrasound image is grabbed from the Sonoscape screen through its VGA output using an A/D converter. These images show cross-sectional slices of the underwater scene. By combining multiple images from different viewpoints, we aim to reconstruct a 3D point cloud of the scene. As the arm precisely controls viewpoints, we can achieve a fairly dense coverage of the target from various angles, though the data will still be incomplete (e.g., the backside of objects remains unobserved) and contain noise (false positives from noise or multi-path reflections).

In our method, converting 2D ultrasound images into a 3D representation is performed in two main steps. First, each ultrasound image is filtered to extract points that are likely to correspond to physical surfaces. Since the acoustic wavefronts propagate radially from the transducer, the 2D image is best interpreted in polar coordinates (r,$\theta$), where r denotes the distance from the transducer along the propagation direction, and $\theta$ denotes the angle. In each angular direction $\theta$, we retain only the first high-intensity echo, and discard any subsequent strong reflections. This way reverberations (multiple reflections from the same surface) and reflections from deeper structures (such as the bottom of the tank) are filtered, and only the surface closest to the transducer in each direction is preserved. Second, the filtered 2D image points are projected into a global 3D coordinate system. Since the ultrasound probe operates with a narrow, planar beam, each image can be considered as a cross-sectional slice of the scanned scene. Given the known pose of the sensor in the global frame, each point $\textbf{p}_{\text {image}} \in \mathbb {R}^3$ from the ultrasound image (expressed in the local sensor frame) is transformed into the global coordinate system using a rigid body transformation:

1 $$\begin{aligned} \textbf{p}_{\text {global}} = \textbf{R} \cdot \textbf{p}_{\text {image}} + \textbf{t} \end{aligned}$$

where $\textbf{R} \in \mathbb {R}^{3\times 3}$ is the rotation matrix representing the sensor orientation, and $\textbf{t} \in \mathbb {R}^3$ is the translation vector describing its position in the global frame. The transformed points from all image frames are combined to form an initial 3D point cloud of the scene.

### Datasets

A critical aspect of training deep learning algorithms is the lack of available large-scale labeled sonar datasets. In our framework the substitutes for such datasets were both synthetic datasets, derived from publicly available 3D models, and real measurements.

PASCAL 3D+ [22] is a database containing on average 3000-3000 3D CAD models within 12 categories, with point-wise classification labels. It is commonly used for object detection and pose estimation tasks. Our primary interests are ships; however, as this dataset contains no such category for part segmentation, we opted for the use of airplanes.

As a complementary dataset, 50 .stl files from meshes of ships, boats, and ship parts or components were collected from publicly available repositories. To generate the 3D point clouds, we selected the centroid of each triangle, as the original vertices were distributed too uniformly. This approach allows us to control the number of points in the point cloud. The size depends on how many points are sufficient to represent the given shape or object. From these, we also created sparse and partial, noisy point clouds to use as input. In the case of sonar data, partial point clouds can mean that the object was scanned from only one side, or that the object lies deep on the seabed.

The ability of the models to generalize from the synthetic data set was tested on real measurements. Some objects (a ship and an airplane) from the complementary dataset were printed using a commercial 3D printer, and their ultrasonic point cloud was acquired as explained in Sect. 2.1.

**Fig. 4:**
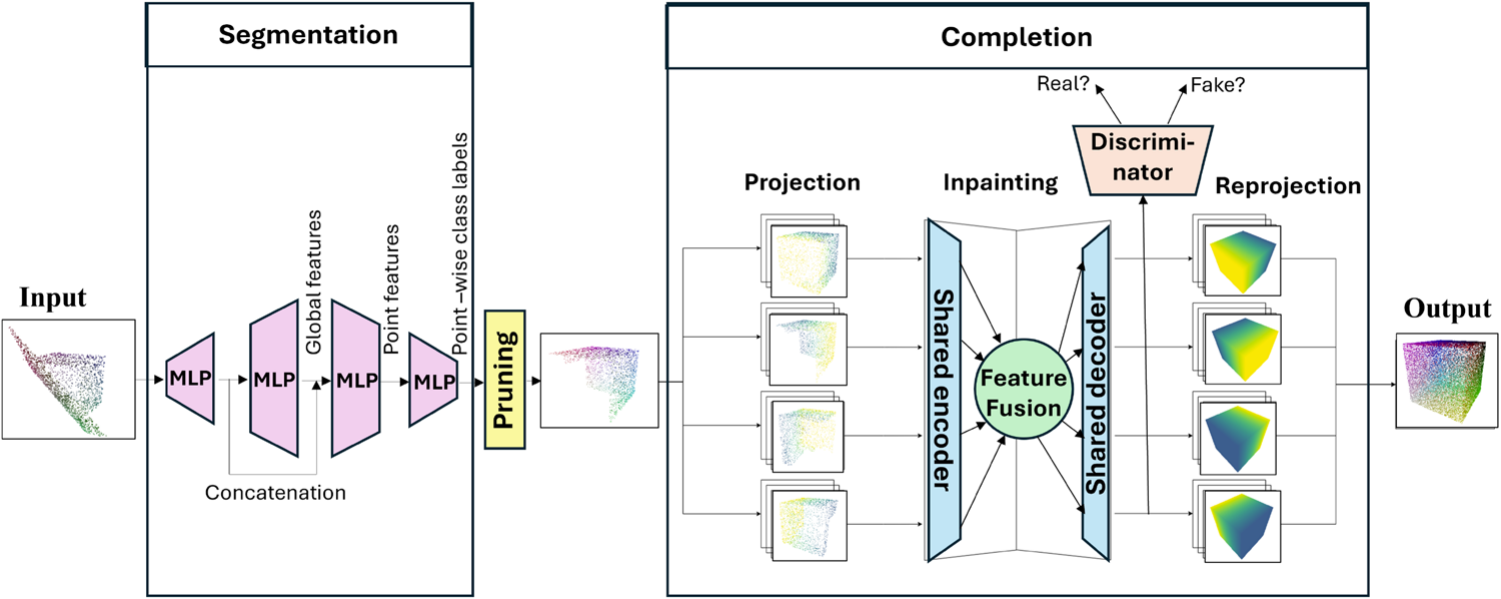
Deep learning pipeline. For illustration purposes, a partial cube lying on a plane is used as the noisy input, which is then run through the PointNet architecture. Global and local features are then used to segment the object of interest (the cube) from the rest of the scene. In the pruning step, only the points belonging to the cube are retained. This low-resolution partial point cloud is then fed into the MVPCC-Net. The first four projections of the mesh are taken and each is fed into a shared encoder for feature extraction. Information from all views is then combined to generate the four inpainted projections via the shared decoder. During training, the generated images are run through the discriminator to determine whether they are real. Finally, the multiple views are combined to produce the 3D point cloud

### Point cloud segmentation using PointNet

A variant of PointNet similar to the segmentation network in the original paper[11] was implemented in Python. It produces per-point labels through a series of MLP layers, a global feature aggregation, and per-point classifiers (see the Segmentation part of Fig. 4).

For preliminary evaluation, we chose the airplanes with “body”, “wing”, “tail”, “engine” and “none” subclasses from the PASCAL 3D+ classes, as they are close in nature to watercrafts, and are also found underwater. In future work we plan to retrain the network for our needs on a larger variety of targets.

Coordinates were shifted with a Gaussian noise to mimic the acoustic nature of the scans. As we expect partial sonar scans from these objects, an optional augmentation step was implemented, slicing the point cloud into two halves along a random plane crossing the centroid of the object. Finally, in order to match the input dimensions of the network, 1024 points were sampled from the point cloud using farthest point sampling [23, 24].

The learning rate was scheduled to decrease by a factor of two every 20 epochs. The categorical cross-entropy loss function was employed to measure the discrepancy between predicted and true class labels, while the Adam optimization algorithm was utilized to update the model parameters.

### Point cloud completion via multi-view projection

At this stage of the pipeline, the input is a segmented point cloud where the points belonging to the object of interest have been identified. Visualizing only the observed points in AR might be sufficient for some awareness, but it can be misleading or less helpful for understanding the full shape and size of the object. Thus, we incorporate a point cloud completion step to predict and fill in the unseen portions, and increase the number of points in the target object geometry.

We adopt a multi-view completion approach inspired by the MVPCC-Net introduced in [19]. The 2D projections of the mesh are processed by a CNN. Features are extracted and fused from all viewpoints, and inpainted, complete projections are generated for each input. These are combined into the complete 3D output. The pipeline is illustrated in the Completion part of Fig. 4.

A set of 4 virtual camera viewpoints around the mesh facing towards the origin are defined. The original implementation used six-channel projections: three for geometry (X, Y and Z) and three for colours (R, G, B). However, as our setup did not include colour information, only three channels were included. For acquiring the low-resolution pairs of the meshes, the slicing, random vertex shifting, and down-sampling steps of Sect. 2.3 were applied. Using the same transformation matrices, projections of these point clouds were also created from the same camera views. The ground truth and the input had a size of $4 \times 256 \times 256 \times 3$, where 4 is the number of viewpoints and 3 is the number of channels.

Apart from the number of channels, the structure of the network remained unchanged, but a new loss function was applied. This was necessary because generative networks are highly sensitive to training data and loss. The composite loss function implemented was

2 $$\begin{aligned} \ell _G = \lambda _1 \ell _{\text {MSE}} + \lambda _2 \ell _{\text {adv}} + \lambda _3 \ell _{\text {Sobel}}, \end{aligned}$$

where $\ell _{\text {MSE}}$ is the mean squared error, $\ell _{\text {adv}}$ is the adversarial loss [25] and $\ell _{\text {Sobel}}$ is the mean squared error between the magnitudes of the target and predicted images, convolved by a Sobel kernel [26]. The weight parameters are $\lambda _1 = 5$, $\lambda _2 = 40$, and $\lambda _3 = 500$. This configuration enforced a smooth background, while keeping the object loss low.

## Results

### Reconstruction and segmentation results

Before implementing the PointNet network, it was unclear how it could predict partial point cloud data when it had only been trained on intact shapes. For this reason, a comparative evaluation was conducted between two PointNet models. The first was trained and validated exclusively on complete point clouds, while the second was trained and validated using the augmented partial point clouds, as described in Sect. 2.3. Both models were subsequently tested on a dataset consisting of cropped point clouds. The dataset was split to 60% training, 10% validation and 30% test folds, with on-the-fly augmentation providing a richer representation of the data.

The overall accuracy did improve after incorporating the slicing of the point cloud (by 0.30), but the class-wise results have revealed a severe imbalance. The less populated ’engine’ and ’none’ labels had extremely low accuracies (even below 0.10). Balanced class weights were calculated based on the aggregated frequency of class labels across objects. After applying these weights during training , the overall accuracy dropped by 0.09, but the mean of the class accuracies improved by 0.07. In this case, even the sparsest ’none’ label achieved an accuracy of 0.54. Table 1 lists the accuracy results of the models. From left to right the networks trained on the complete shapes, partial shapes, and partial shapes with balanced class weights are presented.

**Table 1:** Accuracies of trained networks

	Complete shapes	Partial shapes	Balanced weights
Wing	0.4270	0.8163	0.6136
Body	0.5741	0.9131	0.7389
Tail	0.8356	0.8560	0.9044
Engine	0.0760	0.4667	0.7142
None	0.2924	0.0980	0.5414
Class mean	0.4410	0.6300	0.7025
Overall	0.4953	0.7986	0.7013

**Fig. 5:**
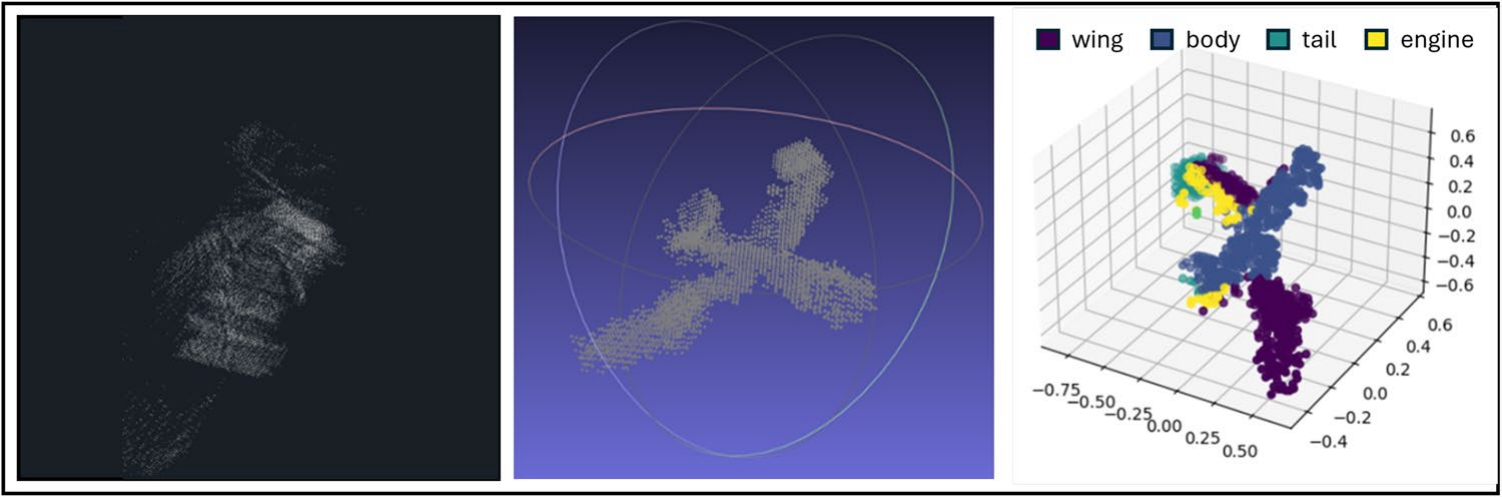
Testing of the segmentation pipeline on a real measurement. Left: the raw measurement of an airplane; middle: the filtered point cloud; right: the output of the segmentation network

In order to test how well the network can generalize to real world data, an ultrasonic measurement from a printed 3D airplane mesh was reconstructed and filtered as explained in Sect. 2.1. The clean point cloud was inferred using the best performing PointNet network, that had been trained on partial shapes, with balanced class weights. The aforementioned steps are illustrated in Fig. 5. It is evident that the body and right wing of the target were accurately predicted; however, there were some discrepancies in labeling the left wing and tail. Nevertheless, the resultant data demonstrates the efficacy of the trained PointNet network in generalizing from synthetic data to real-world measurement, subsequent to the implementation of the applied augmentations.

### Point cloud completion results

**Fig. 6:**
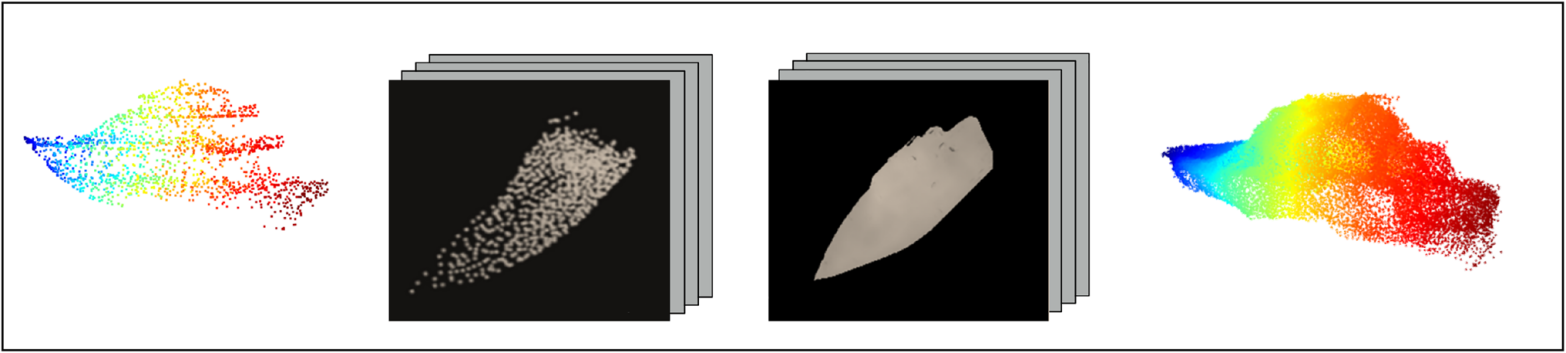
Testing of the completion pipeline on a synthetic input with a missing bottom part. The sequence of images, from left to right, comprises: the sparse, partial point cloud; projections of the input; inpainted projections; re-projections

A training-to-validation split ratio of 0.9 to 0.1 was employed on the synthesized dataset. Experiments were conducted using sparse input point clouds consisting of 800 or 2500 points. Each input–target pair was trained six times with varying combinations of loss functions to observe the visual impact on the generated outputs. The most effective configuration involved a composite loss function comprising object loss (weight = 3), mean squared error loss (weight = 2), adversarial loss (weight = 10), and feature matching loss (weight = 10), with an input point cloud size of 2500 points. Under these conditions, the model produced high-quality visual projections, with finer structural details beginning to emerge after approximately 50 training epochs. A representative result for a training sample with similarly sampled input is shown in Fig. 6. The re-projection of the data generates a denser point cloud, preserving the overall form of the ship, with the lower portion of the hull nearing completion.

**Fig. 7:**
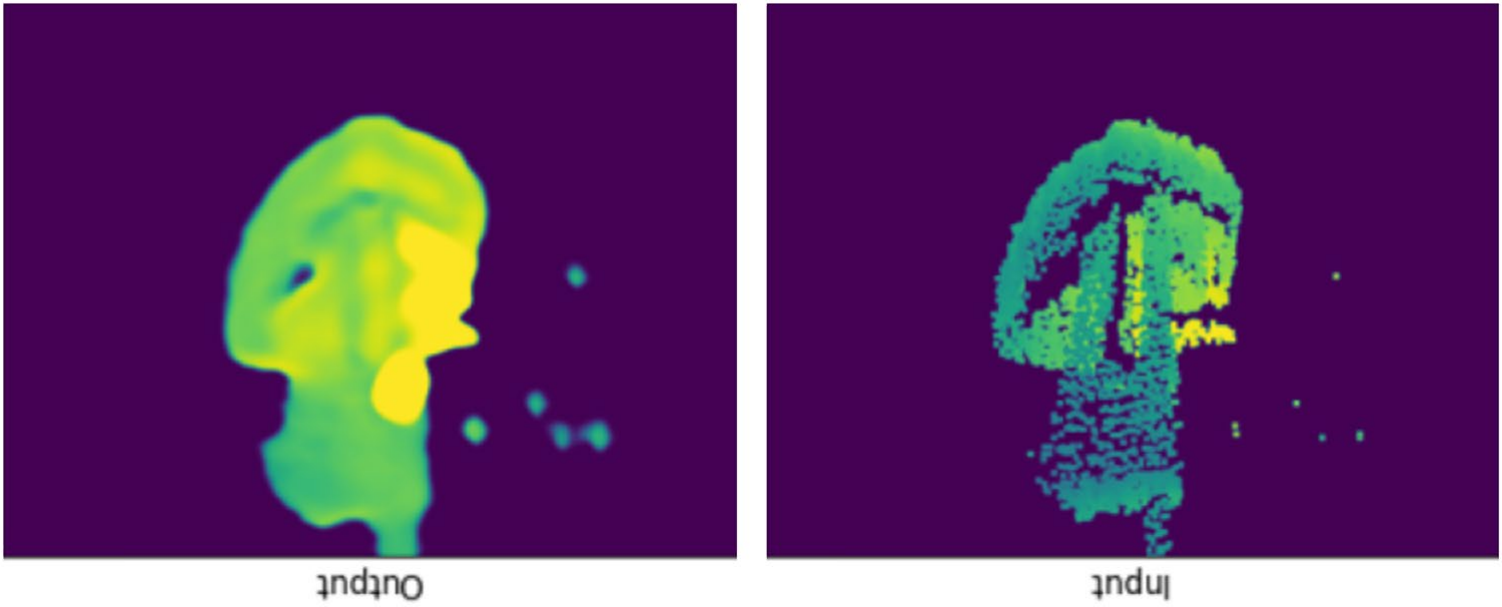
Testing of the completion pipeline on a real measurement input

It is evident from our tests that finding a descriptive loss, promoting a smooth image from a salt-and-pepper-type input, while maintaining a constant background and keeping edges and fine details, is a challenge. When inferring a real measurement of one of the boat models, we arrived to the same conclusion. As shown in Fig. 7, the model promoted large constant areas, loosing fine details.

## Discussion

In this work, we explored how augmented reality technologies, supported by deep learning, can significantly enhance human spatial awareness in challenging environments. The review of the literature has been supported by testing these methods in a constructed framework. The underwater, obscured vision is augmented through reconstructed and interpreted sonar data. The system combined a robotic ultrasound scan, point cloud generation, PointNet segmentation (retrained for the domain), and a multi-view completion network to create humanly interpretable sonar point clouds. Experimental results demonstrated that our approach can segment and identify targets in noisy sonar point clouds and produce plausible complete and denser models, improving the understanding of the user about the scene.

The research also shed light on challenges, such as the need for robust training data, appropriate measures of the visual properties of point clouds, and system calibration. Future work requires an expansion of the training datasets. For synthetic data, this means including more object classes and incorporating speckle noise to better resemble sonar data. To fine-tune the trained networks, sonar scans of objects with known shapes in different environments must be acquired. Initial steps have also been taken in visualization on AR glasses, but the currently available tools do not support underwater testing of the pipeline.

In conclusion, the synergy of AR and AI-driven spatial computing holds great promise for bridging the gap between humans and environments that are currently beyond our natural sensory capabilities. By converting rich sensor data into human-friendly augmented experiences, we enable users to achieve a level of situational awareness previously unattainable. The ongoing advancements in both hardware (sensors, AR displays) and algorithms (deep learning for 3D understanding) will further pave the way toward more immersive and reliable spatial awareness systems.

## References

[1] Carbonell-Carrera C, Bermejo-Asensio LA. Augmented reality as a digital teaching environment to develop spatial thinking. Cartogr Geogr Inf Sci. 2017;44(3):259–70. 10.1080/15230406.2016.1275718.

[2] Guntur J, et al. Can augmented reality improve problem-solving and spatial skill? J Phys Conf Ser. 2020;1581(1):012063.

[3] Goldstein P. How AR firefighting masks improve situational awareness. StateTech Magazine. 2019.https://statetechmagazine.com/article/2019/11/how-ar-firefighting-masks-improve-situational-awareness.

[4] Office of naval research (ONR). US navy divers get augmented vision display. Marine technology news. 2025. https://www.marinetechnologynews.com/news/navy-divers-augmented-vision-584643.

[5] Teixeira PV, Fourie D, Kaess M, Leonard JJ. Dense, sonar-based reconstruction of underwater scenes. In: 2019 IEEE/RSJ international conference on intelligent robots and systems (IROS). 2019. pp. 8060-66. 10.1109/IROS40897.2019.8968071.

[6] Hu S, Liu T, et al. Underwater rescue target detection based on acoustic images. Sensors. 2024;24(6):1780. 10.3390/s24061780. 38544042 10.3390/s24061780 PMC10974114

[7] Maturana D, Scherer S. Voxnet: a 3d convolutional neural network for real-time object recognition. In: Proceedings IEEE/RSJ international conference on intelligent robots and systems (IROS). 2015. pp. 922–28.

[8] Dai A, Qi CR, Nießner M. Shape completion using 3d-encoder-predictor CNNS and shape synthesis. In: Proceedings IEEE conference on computer vision and pattern recognition (CVPR). 2017. pp. 6545–54.

[9] Lei H, Akhtar N, Mian A. Octree guided CNN with spherical kernels for 3d point clouds. In: Proceedings of the IEEE/CVF conference on computer vision and pattern recognition (CVPR). 2019. pp. 2017–26. 10.1109/CVPR.2019.00212.

[10] Zhai X, et al. Sparsepipe: parallel deep learning for 3d point clouds. In: Proceedings of the IEEE/CVF international conference on computer vision. IEEE; 2021. pp. 13307–316.

[11] Qi CR, Su H, Mo K, Guibas LJ. Pointnet: deep learning on point sets for 3d classification and segmentation. In: Proceedings IEEE conference on computer vision and pattern recognition (CVPR). 2017. pp. 77–85.

[12] Qi CR, Yi L, Su H, Guibas LJ. Pointnet++: deep hierarchical feature learning on point sets in a metric space. In: Advances in neural information processing systems (NIPS). vol. 30. 2017.

[13] Kim J, Cho H, Lee M, Yu SC. Underwater-sonar-image-based 3d point cloud reconstruction for high data utilization and object classification using a neural network. Electronics. 2020;9(11):1763. 10.3390/electronics9111763.

[14] Yuan W, Khot T, Held D, Mertz C, Hebert M. PCN: point completion network. In: Proceedings international conference on 3D vision (3DV). 2018. pp. 728–37.

[15] Pan L, Chen X, Cai Z, Xu K, Tong X, Liu L. Variational relational point completion network. In: Proceedings of the IEEE/CVF conference on computer vision and pattern recognition (CVPR). IEEE; 2021. pp. 8520–29. 10.1109/CVPR46437.2021.00842.

[16] Pan L. ECG: edge-aware point cloud completion with graph convolution. IEEE Robot Auto Lett. 2020;5(3):4392–8. 10.1109/LRA.2020.2994483.

[17] Park C, Jeong Y, Cho M, Park J. Fast point transformer. In: Proceedings of the IEEE/CVF conference on computer vision and pattern recognition. 2022. pp 16949–58.

[18] Su H, Maji S, Kalogerakis E, Learned-Miller E. Multi-view convolutional neural networks for 3d shape recognition. In: Proceedings IEEE international conference on computer vision (ICCV). 2015. pp 945–53.

[19] Ibrahim Y, Benedek C. MVPCC-Net: multi-view based point cloud completion network for MLS data. Image Vis Comput. 2023;134:104675. 10.1016/j.imavis.2023.104675.

[20] Yang B, Luo W, Urtasun R. Pixor: real-time 3d object detection from point clouds. In: Proceedings of the IEEE conference on computer vision and pattern recognition (CVPR). IEEE; 2018. pp 7652–60. 10.1109/CVPR.2018.00799.

[21] Biasutti P, Lepetit V, Aujol J-F, Brédif M, Bugeau A. LU-Net: an efficient network for 3D lidar point cloud semantic segmentation based on end-to-end-learned 3D features and U-net. 2019. arXiv:1908.11656.

[22] Xiang Y, Mottaghi R, Savarese S. Beyond pascal: a benchmark for 3d object detection in the wild. In: 2014 IEEE winter conference on applications of computer vision (WACV). IEEE; 2014. pp 75–82. 10.1109/WACV.2014.6836101.

[23] Han M, Wang L, Xiao L, Zhang H, Zhang C, Xu X, Zhu J. Quickfps: architecture and algorithm co-design for farthest point sampling in large-scale point clouds. IEEE Trans Comput Aided Des Integr circuits Syst. 2023.

[24] Qi CR, Yi L, Su H, Guibas LJ. Pointnet++: deep hierarchical feature learning on point sets in a metric space. In: Advances in neural information processing systems (NeurIPS), vol. 30. 2017.

[25] Goodfellow IJ, Pouget-Abadie J, Mirza M, Xu B, Warde-Farley D, Ozair S, Courville A, Bengio Y. Generative adversarial networks. 2014. arXiv preprint arXiv:1406.2661. 10.48550/arXiv.1406.2661.

[26] Shan G, Jia S. Convolution-based probability gradient loss for semantic segmentation. 2024. arXiv preprint, submitted 10 April 2024. arXiv:2404.06704v1.

